# In vitro labelling and detection of mesenchymal stromal cells: a comparison between magnetic resonance imaging of iron-labelled cells and magnetic resonance spectroscopy of fluorine-labelled cells

**DOI:** 10.1186/s41747-017-0010-9

**Published:** 2017-06-29

**Authors:** Stefania Rizzo, Francesco Petrella, Ileana Zucca, Elena Rinaldi, Andrea Barbaglia, Francesco Padelli, Fulvio Baggi, Lorenzo Spaggiari, Massimo Bellomi, Maria Grazia Bruzzone

**Affiliations:** 1 Department of Radiology, European Institute of Oncology, via Ripamonti 435, 20141 Milan, Italy; 2 Department of Thoracic Surgery, European Institute of Oncology, Milan, Italy; 3 Scientific Department, Neurological Institute IRCCS “Carlo Besta”, Milan, Italy; 4 Neuroimmunology and Neuromuscular Diseases Unit, Neurological Institute IRCCS “Carlo Besta”, Milan, Italy; 5 Department of Oncology and Hemato-Oncology, Università degli Studi di Milano, via Festa del Perdono 7, 20142 Milan, Italy; 6 Department of Neuroradiology, Neurological Institute IRCCS “Carlo Besta”, Milan, Italy

**Keywords:** Mesenchymal stromal cells (MSCs), Superparamagnetic iron oxide (SPIO), Perfluorocarbon (PFC), Cell labelling, Cell tracking

## Abstract

**Background:**

Among the various stem cell populations used for cell therapy, adult mesenchymal stromal cells (MSCs) have emerged as a major new cell technology. These cells must be tracked after transplantation to monitor their migration within the body and quantify their accumulation at the target site. This study assessed whether rat bone marrow MSCs can be labelled with superparamagnetic iron oxide (SPIO) nanoparticles and perfluorocarbon (PFC) nanoemulsion formulations without altering cell viability and compared magnetic resonance imaging (MRI) and magnetic resonance spectroscopy (MRS) results from iron-labelled and fluorine-labelled MSCs, respectively.

**Methods:**

Of MSCs, 2 × 10^6^ were labelled with Molday ION Rhodamine-B (MIRB) and 2 × 10^6^ were labelled with Cell Sense. Cell viability was evaluated by trypan blue exclusion method. Labelled MSCs were divided into four samples containing increasing cell numbers (0.125 × 10^6^, 0.25 × 10^6^, 0.5 × 10^6^, 1 × 10^6^) and scanned on a 7T MRI: for MIRB-labelled cells, phantoms and cells negative control, T1, T2 and T2* maps were acquired; for Cell Sense labelled cells, phantoms and unlabelled cells, a ^19^F non-localised single-pulse MRS sequence was acquired.

**Results:**

In total, 86.8% and 83.6% of MIRB-labelled cells and Cell Sense-labelled cells were viable, respectively. MIRB-labelled cells were visible in all samples with different cell numbers; pellets containing 0.5 × 10^6^ and 1 × 10^6^ of Cell Sense-labelled cells showed a detectable ^19^F signal.

**Conclusions:**

Our data support the use of both types of contrast material (SPIO and PFC) for MSCs labelling, although further efforts should be dedicated to improve the efficiency of PFC labelling.

## Key Points

- SPIO-labelled cells are viable and MRI-detectable at all dilutions tested
- PFC-labelled cells are viable and MRS-detectable if > 0.5 × 10^6
- Detection of MSCs might consider multimodal approaches including SPIO and PFC compounds

## Introduction

The persistent tissue and organ shortage has led to the emergence of regenerative medicine. This interdisciplinary field involving biology, medicine and engineering aims to repair, replace, maintain or enhance tissue and organ functions by means of cell therapy [1].

Among the various stem cell populations used for cell therapy, adult mesenchymal stromal cells (MSCs) have emerged as a major new technology with many potential clinical applications [2]. MSCs are a population of undifferentiated multipotent adult cells that naturally reside within the human body and are generally defined as plastic-adherent, fibroblast-like cells possessing extensive self-renewal properties and the potential to differentiate in vivo and in vitro into a variety of mesenchymal lineage cells [3].

MSCs have the ability to migrate and engraft at sites of inflammation and injury in response to cytokines, chemokines and growth factors. They can also exert local reparative effects through trans-differentiation into tissue-specific cell types or via the paracrine secretion of soluble factors with anti-inflammatory and wound-healing activities [4].

There is a specific need to track these cells after transplantation, evaluate different methods of implantation, monitor cell migration within the body and quantify cell accumulation at the target site [2].

Magnetic resonance imaging (MRI) has emerged as an excellent method for tracking cells both in vivo and in vitro [5]. Many cell-tracking studies have used superparamagnetic iron oxide (SPIO) nanoparticle-based contrast agents to label cells for detection with MRI [6–8], while others have used perfluorocarbon (PFC) nanoemulsion formulations [9–11]. The ^19^F nucleus is particularly suitable for labelling as its relative MRI sensitivity is only 17% lower than that of hydrogen nucleus [12]. Due to the absence of background ^19^F signal in host tissue, fluorine contrast agents can not only localise, but also quantify the cells delivered by the direct quantification of the probe through a known reference phantom [13]. However, no consistent results on MSC detection and MRI tracking have been obtained so far.

The purpose of this study was to assess if MSCs can be labelled with SPIO nanoparticles and PFC nanoemulsion formulations without altering cell viability and compare MRI findings from iron-labelled MSCs with magnetic resonance spectroscopy (MRS) findings from fluorine-labelled MSCs.

## Methods

### Rat mesenchymal stem cell culture

StemPro® Rat Alk Phos Expressing MSCs were purchased from ThermoFisher Scientific (cat. no. R7789120) and cultured in α-Minimum Essential Medium, with nucleosides and GlutaMAX™ (ThermoFisher Scientific, cat. no. 32571), supplemented with 10% fetal bovine serum (ThermoFisher Scientific, cat. no. 10270) and 1% penicillin-streptomycin solution 100× (Euroclone, cat. no. ECB3001D). MSCs were isolated from bone marrow of transgenic Fischer 344 rats expressing the human placental alkaline phosphatase (hPAP) gene. The medium was changed every third day and MSCs were maintained at 37 °C, 5% CO_2_.

### SPIO labelling and ^19^F labelling

MSCs (passage 5) were treated with trypsin-EDTA (cat. no. Euroclone, ECB3052D) and centrifuged at 300 g for 5 min. MSCs count (expressed as number of cells/mL) and cell viability (calculated as number of viable cells/number of dead cells + number of viable cells and expressed as %) were evaluated with the trypan blue exclusion method. 2 × 10^6^ viable MSCs were seeded in a tissue culture flask (75 cm^2^) for labelling with Molday ION Rhodamine-B (MIRB, BioPal Inc, Worcester, MA, USA), SPIO nanoparticles conjugated with Rhodamine B, that can be visualised by fluorescent imaging. MIRB has a colloidal size of 35 nm, a zeta potential of ~ +31 mV and an iron concentration of 2 mg/mL. MIRB was added to MSC culture at a concentration of 50 μg/mL in 6 mL culture medium for 24 h at 37 °C. 2 × 10^6^ MSCs were labelled with Cell Sense (CS-ATM DM Green), a PFC emulsion conjugated with a green fluorescent dye, commercially obtained from Celsense Inc. (Pittsburgh, PA, USA). Cell Sense has a total fluorine content of 120 mg/mL. MSCs were incubated with Cell Sense at a concentration of 10 mg/mL in 6 mL of culture medium for 24 h at 37 °C. Unlabelled control MSCs (2 × 10^6^ cells) were incubated in 6 mL of culture medium for 24 h at 37 °C. After incubation, the culture medium was aspirated and all the MSCs cultures were washed twice with phosphate-buffered saline (PBS) to remove extracellular labelling agents. MIRB-MSCs, Cell Sense-MSCs and control MSCs were then treated with trypsin-EDTA and centrifuged at 300 g for 5 min; cell count and cell viability were evaluated with the trypan blue exclusion method. 10^4^ labelled cells (MIRB-MSCs and Cell Sense-MSCs) were resuspended in culture medium and seeded on a chamber slide. The culture medium was aspirated, cells were washed twice with PBS and fixed with 4% paraformaldehyde (PFA) for 15 min and stored at 4 °C. Cells were permeabilised with PBS-Triton 0,1% for 10 min and nuclei were stained with DAPI (Thermo Fisher Scientific, cat. no. D1306).

Images were acquired by confocal microscopy (Eclipse TE2000-E microscope equipped with EZ-C1 scan-head; Nikon) using a 20× (NA 0.85) objective to evaluate labelling efficiency; structured illumination microscopy (SIM) with 100× Apo-TIRF (NA 1.49) objective (Nikon) was used to assess MIRB and Cell Sense cytoplasmic distribution. SIM achieves a lateral resolution of 100–130 nm [14].

The number of labelled MSCs (visualised by green fluorescence for Cell Sense-labelled MSCs and by red fluorescence for MIRB-labelled MSCs) and the number of analysed cells (number of cell nuclei, blue stained by DAPI) were evaluated using the ImageJ Software Cell-Counter plug-in. The percentage of MIRB-labelled and Cell Sense-labelled MSCs was then calculated.

### Cell phantom preparation

Labelled MSCs were fixed in 4% paraformaldehyde and divided into four samples containing increasing numbers of cells (0.125 × 106, 0.25 × 106, 0.5 × 106, 1 × 106). MSC samples were prepared by centrifugation at 300 g for 5 min and each microcentrifugation tube was filled up with low-melting 1% agarose (Bio-Rad, 162–0017). Cell phantoms of unlabelled cells and a positive control with MIRB and Cell Sense at the same concentrations used for cell labelling were prepared.

MSCs were expanded and labelled in culture medium at physiological pH. Before MRI/MRS analysis, cells were fixed in 4% PFA solution pH 7.4 and washed in PBS pH 7.4; therefore, the pH was always maintained at physiological level.

### MRI/MRS characteristics and features

The acquisitions were performed on a horizontal 7T MRI scanner (Bruker BioSpec 70/30, Ettlingen, Germany) equipped with a gradient system reaching a maximum amplitude of 440 mT/m. Both MRI for MIRB-labelled cells and MRS for Cell Sense-labelled cells were acquired by a double nuclei (^1^H/^19^F) volume resonator with an inner diameter of 72 mm.

Since contrast agents relaxivity depends on extrinsic factors such as applied field and temperature, all MRI acquisition were performed at the magnet bore temperature, strictly maintained constant at 18 °C.

T1, T2 and T2* MRI maps were acquired with the same geometry (one 1-mm coronal slice including the cells pellet) on MIRB-labelled cells, the MIRB phantom and cell negative control. T2-weighted images with 0.7-mm slices in axial, sagittal and coronal geometries (with respect to the magnet reference frame) were also acquired to perform relaxometry maps reference images by visualising the hypo-intensity corresponding to the labelled cells pellets.

T1 mapping was based on a rapid acquisition with relaxation enhancement (RARE) sequence with a RARE factor of 2; 28 images, with a different repetition time (TR) in the range of 25–15,000 ms, were acquired; echo time (TE) was set to 4.6 ms, in-plane resolution was 313 × 313 μm^2^, the slice thickness was 1 mm, the number of excitations was 2; the total acquisition time was 14 min. A region of interest (ROI) was then selected corresponding to the sample signal and signal intensity (SI) plotted versus the image TR. The curve was fitted by the function

$$ S I= A+ C\left(1- \exp -\frac{ T R}{T_1}\right) $$

where A is the absolute bias and C the maximal SI.

T2 mapping was based on a multi-slice multi-echo (MSME) sequence with a series of 200 echo images a with different TE (range 5–937.5 ms); TR was set to 5000 ms, in-plane resolution was 313 × 313 μm^2^, the slice thickness was 1 mm, the number of excitations was 28; the total acquisition time was 13 h. A ROI was then selected corresponding to the sample signal and SI was plotted versus the image TE. The curve was fitted by the function

$$ S I= A+ C\left( \exp -\frac{ T E}{T_2}\right) $$

where A is the absolute bias and C the maximal SI.

T2* mapping is based on a multiple gradient echo (MGE) sequence with a series of 500 echo images with a different TE (range of 2–807 ms); TR is set to 5000 ms, in-plane resolution was 313 × 313 μm^2^, the slice thickness was 1 mm, the number of excitations was 5; the total acquisition time was 50 min. A ROI was selected corresponding to the sample signal and its SI was plotted versus the image TE. The fitting was performed by the T2 maps analysis function.

All curves fittings were performed by the built-in least squares minimisation Levenberg-Marquardt routines of OriginLab (Northampton, MA, USA, http://www.originlab.com/) software. Reported errors are the fit’s standard deviations.

A T2-weighted RARE sequence was performed with the following parameters: Hermite pulse centred at 300 MHz with 2 kHz bandwidth, TR = 3100 ms, TE = 54 ms, flip angle (FA) = 90°, slice thickness = 0.7 mm, in-plane resolution = 156 μm × 156 μm^2^. The total acquisition time was 1 min 36 s.

Non-localised single-pulse ^19^F-MRS sequences (Block pulse centered at 209 MHz with 14 kHz bandwidth, FA 90°, TR 20 s, 100 excitations, total acquisition time 33 min) were acquired on Cell Sense-labelled cells, Cell Sense phantom and cell negative control to ensure the detectability of each sample. For each sample, two acquisition sessions were performed one week apart.

## Results

### SPIO- and ^19^F-labelled cells viability and labelling efficiency

**Fig. 1:**
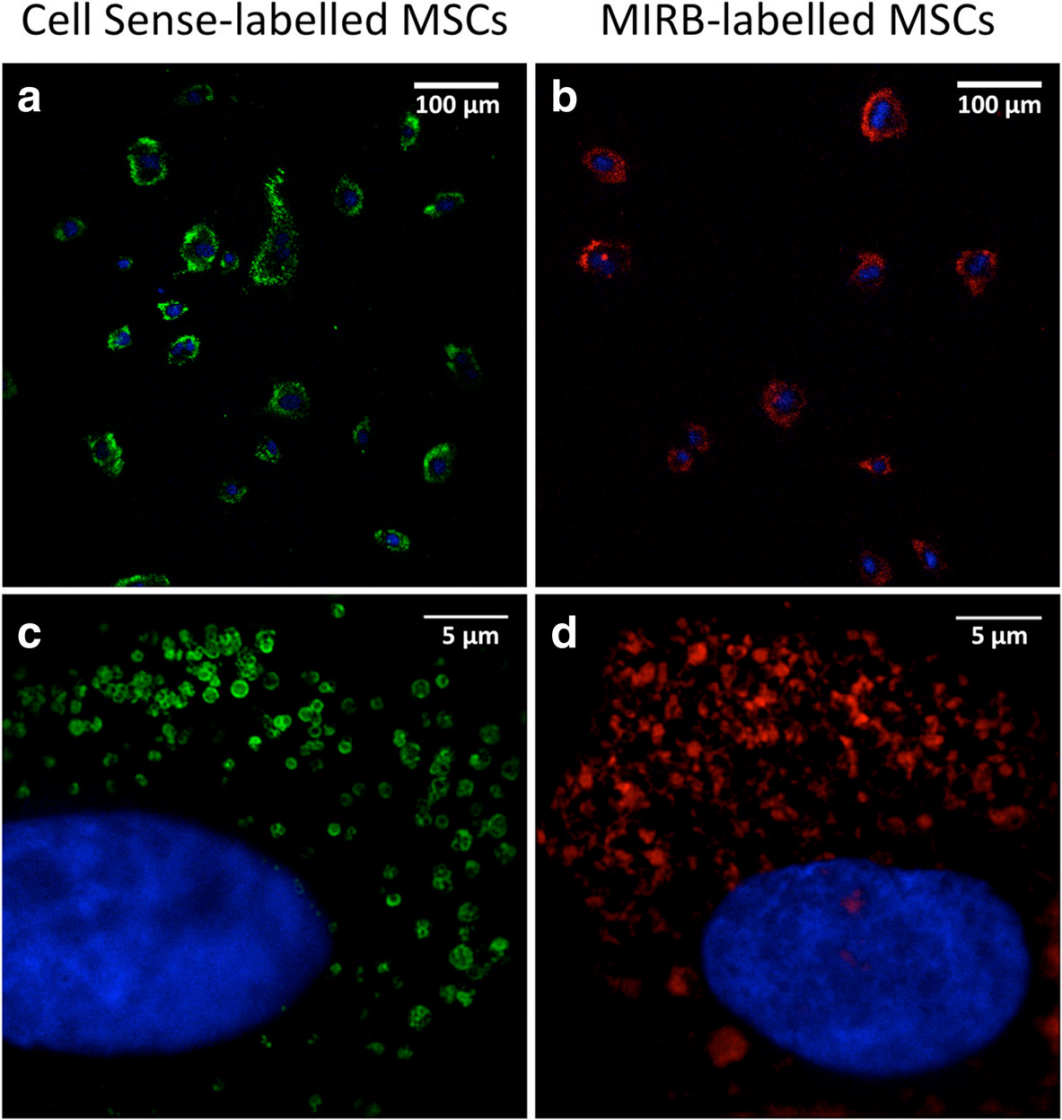
Cell Sense-labelled MSCs and MIRB-labelled MSCs. MSCs were labelled with Cell Sense (10 mg/mL, green fluorescence; **a** and **c**) or with MIRB (50 μg/mL, red fluorescence; **b** and **d**). Nuclei were counterstained with DAPI (*blue*). Images were acquired by confocal microscopy (**a** and **b**) and by structured illumination microscopy (**c** and **d**). Scale bars: **a** and **b**, 100 μm; **c** and **d**, 5 μm

MIRB and Cell Sense uptake was assessed by confocal microscopy (Fig. 1a and b) to evaluate the labelling efficiency; cytoplasmic distribution in MSCs was observed through structured illumination microscopy (Fig. 1c and d), which provides a better resolved image and a more precise MIRB and Cell Sense localisation in MSC.

Cell viability was evaluated to exclude any toxic effect of SPIO or Cell Sense. MSC viability was not significantly different from the control group (86.8% versus 89.8%); 99.2% of MSCs were found to have fluorescent (red) signals from the rhodamine dye (MIRB) molecules distributed in the cytoplasm.

Similarly, after 24 h of incubation with Cell Sense, this fluorescently labelled tracer (detected in green) was found in 94.6% of MSCs, localised in vesicles diffusely distributed in the cytoplasm. We observed a slight decrease in cell viability (83.6%) compared with control MSCs cultures (89.8%).

### MRI of SPIO-labelled cells

**Fig. 2:**
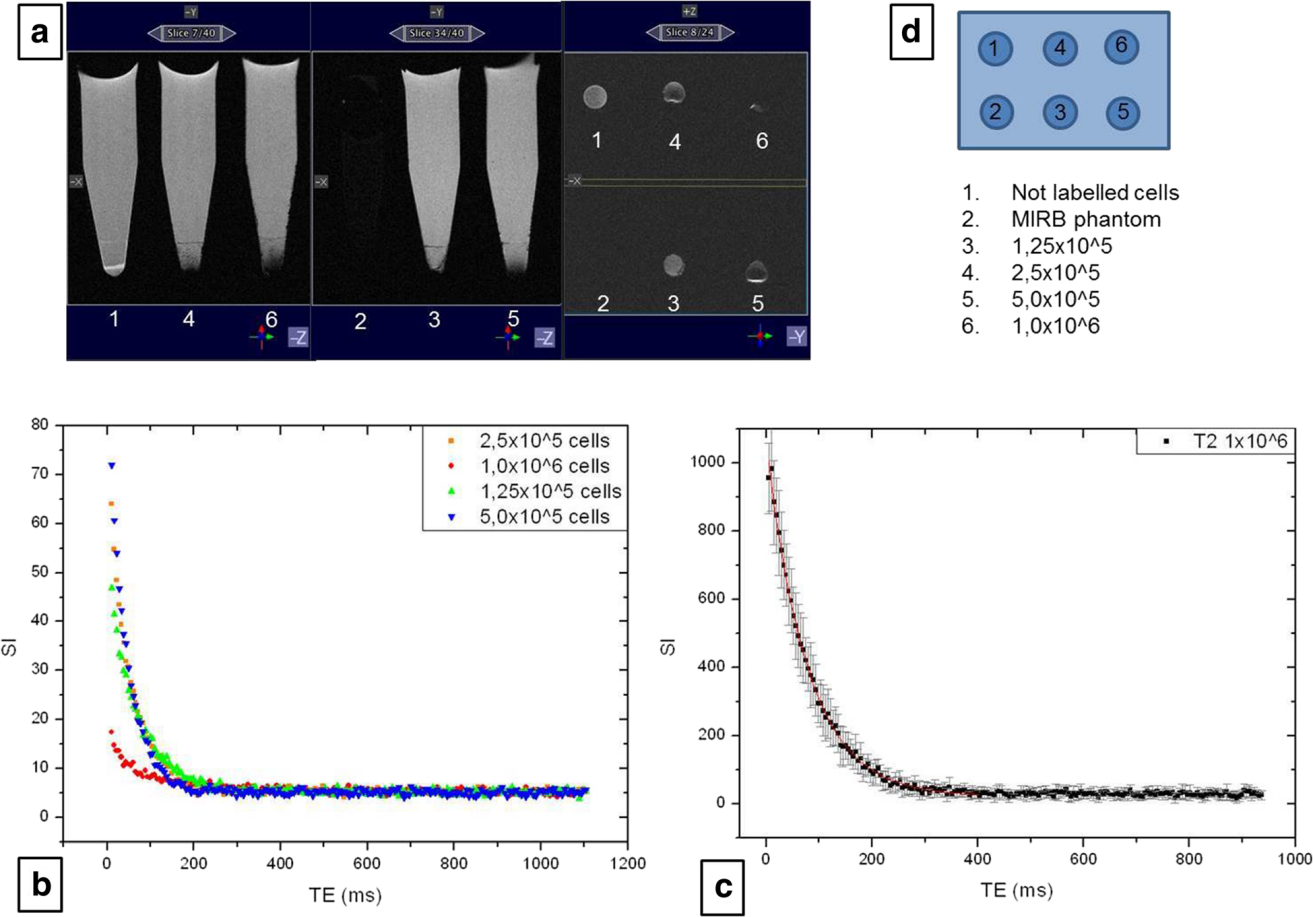
**a** Coronal and axial T2-weighted images of six samples containing unlabelled cells (1), MIRB (2) and different amounts of MIRB labelled cells (3, 4, 5, 6). **b** T2 decays of the labelled cells samples. **c** T2 data fitting of the 1 × 10^6^ cells pellet. **d** Samples positions (coronal view) during MRI acquisitions

MIRB showed the following relaxation parameters: T1 = 559 ± 5 ms, T2 = 13.4 ± 0.8 ms, T2* = 3.6 ± 0.5 ms. T1, T2 and T2* values for unlabelled cells were 2041 ± 12 ms, 132 ± 0.4 ms and 4.6 ± 0.1 ms, respectively. Table 1 lists the relaxation times of the MIRB phantom, unlabelled and MIRB-labelled cells. Because the MIRB-labelled cells T2* decay times is too short and affected by agar susceptibility artefacts, meaningful curve fit cannot be obtained. T2-weighted images showed a hypointense signal corresponding to labelled cells pellet regions, visible in all samples with different numbers of cells (Fig. 2).

**Table 1:** MIRB phantom cells, unlabelled cells and MIRB-labelled cells relaxometry results. Reported errors are the fit’s standard deviations

Cell number	T1 relaxation time (ms)	T2 relaxation time (ms)	T2* relaxation time (ms)
0.125 E6	2128 ± 181	65.6 ± 0.8	
0.25 E6	1493 ± 111	50.5 ± 0.5	
0.5 E6	2000 ± 280	41.9 ± 0.4	
1 E6	2500 ± 44	74.6 ± 0.5	
Unlabelled cells	2041 ± 12	132 ± 0.4	4.6 ± 0.1
MIRB phantom	559 ± 5	13.4 ± 0.8	3.6 ± 0.5

### MRS of ^19^F-labelled cells

**Fig. 3:**
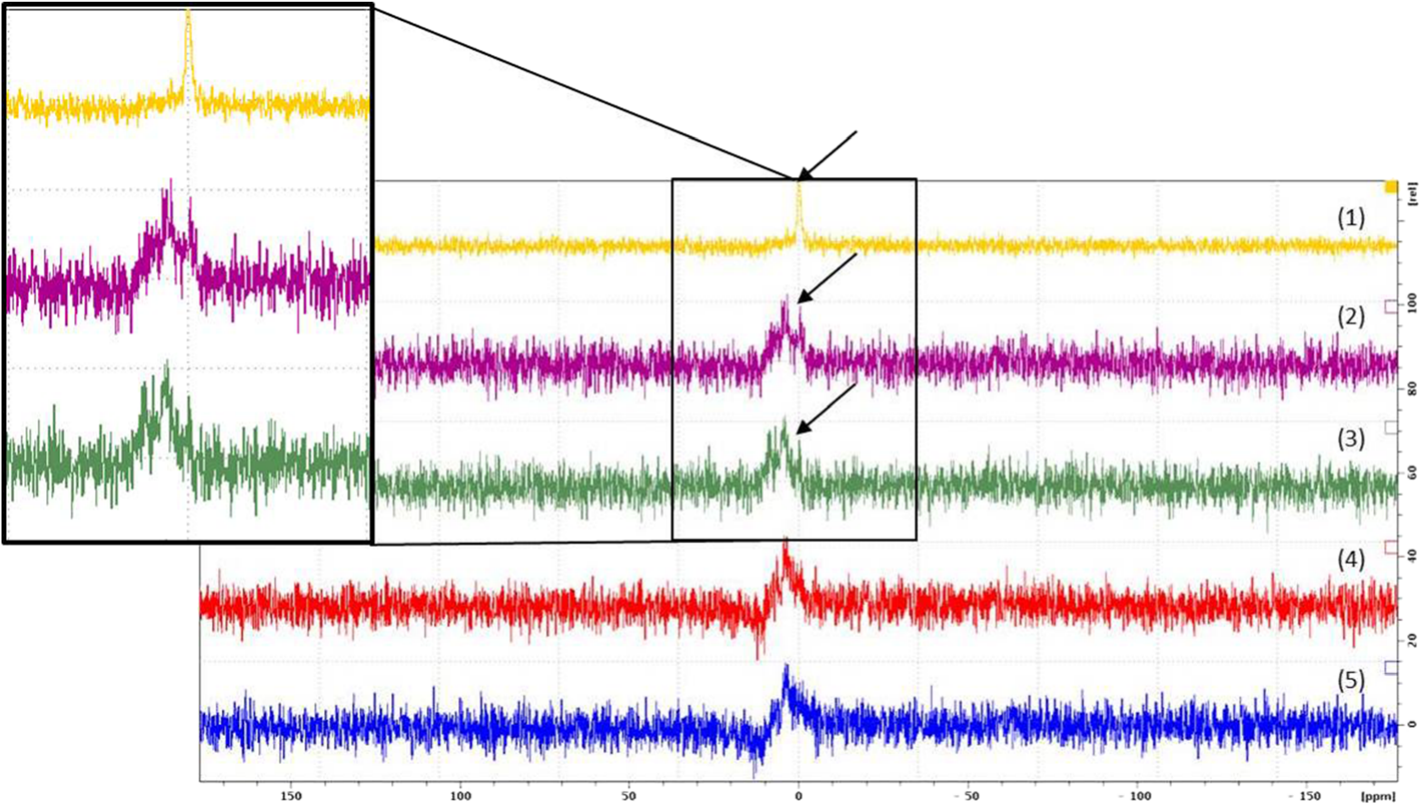
^19^F MRS of five samples containing Cell Sense (1) and different amounts of Cell Sense-labelled cells: 1 million (2), 5 × 10^5^ (3), 2.5 × 10^5^ (4), 1.25 × 10^5^ (5). Black arrows indicate the Cell Sense fluorine signal, while the wide peak at −5 ppm is due to the resonator coil itself

Only pellets containing 1 million and half a million of Cell Sense-labelled cells showed a detectable signal, although with a very low intensity in comparison with noise (Fig. 3). While negative control cells did not show any signal, the Cell Sense phantom showed its characteristic ^19^F peak.

The two spectra acquired one week apart did not show any shift attributable to a pH variation.

## Discussion

Stem cells are being intensively studied for tissue infusion or transplantation into tissue for purposes of repair, revascularisation and other therapeutic measures [15–17]. However, the serial visualisation and tracking of transplanted stem cells, including their quantification at the site of injury and migration or retention in other sites, remain unresolved issues. Indeed, there is still some uncertainty on the safety of these cells [18] and further pre-clinical validation is necessary before the clinical application of stem cell therapy.

A safe, non-invasive and repeatable imaging technique able to track the injected stem cells in vivo could solve some of the current issues, but no single imaging modality can currently yield all the information required to monitor stem cell labelling and in vivo tracking.

As MRI is non-invasive and offers high spatial resolution (ranging from 50 μm in animals and up to 300 μm in whole body clinical scanners), it is a popular choice for in vivo cell tracking in pre-clinical and clinical studies [19]. For MRI tracking, stem cells need to be enriched with a contrast agent providing a sufficient positive or negative difference in SI to distinguish the cells from the image background. Contrast agents containing SPIO nanoparticles have been the preferred agent for short-term stem cell tracking, due to the pronounced signal decrease that even small amounts of these contrast media can create, owing to the so-called 'blooming artefact'.

Thanks to this negative contrast effect, the present study detected all the MIRB-labelled cell samples by a hypointense signal on T2-weighted images. The resolution of these images was compatible with in vivo experiments (acquisition time was 1 min 36 s, compatible with anaesthetised animal’s stay in the magnet) and can also depict the hypointensity related to pellets containing the lowest number of cells (0.125 × 10^6^).

This study reports only mono-exponential relaxometry results. A bi-exponential model to fit the curves reported in Fig. 2b and c can be ruled out because previous in vitro studies [20, 21], testing bi-exponential fitting functions in T2 decays, demonstrated a negligible component with a very short time constant and a predominant component with a time constant comparable to the mono-exponential fitting relaxation time.

Furthermore, it can be assumed that our MIRB sample results were not affected by temperature dependence: in agreement with the literature [22] and as demonstrated by acquisitions repeated one week later, the proprieties of SPIO nanoparticles used as T2 contrast agents depend on the size of the nanoparticle core and are less influenced by temperature than T1 agents [22].

The SPIO negative contrast enhancement allows for detecting even very small numbers of labelled cells but can be confounded with other sources of magnetic susceptibility effects, such as bleeds, blood vessels or air, depending on the anatomical structure under evaluation. As future pre-clinical applications may include lung evaluation for MSCs infusion in repair processes, such as broncho-pleural fistulae [23], and assessing cell migration into the lungs, we also labelled MSCs with PFC nanoemulsions, which can be detected with ^19^F MRI and MRS [9, 10].

Moreover, labelled MSCs stably express the hPAP gene, thus allowing cell detection by classical histochemical methods, making this gene a useful tracking tool for further in vivo MRI validation.

Our study labelled MSCs with a PFC emulsion conjugated with BODIPY dye with a slight decrease in cell viability compared with the control (83.6% versus 89.8%), but only pellets containing 1 × 10^6^ and 0.5 × 10^6^ of Cell Sense-labelled cells showed a detectable signal. Compared with labelling and tracking with metal-based contrast agents, ^19^F labelling is less sensitive, therefore a relatively large amount of ^19^F, or a large number of labelled cells, must accumulate in order to generate a signal-to-noise ratio sufficient for tracking.

Our data support the use of both types of contrast media (SPIO and PFC) for MSCs labelling, although further efforts should be dedicated to improve the efficiency of PFC labelling.
